# Violations of Hyperscaling in Finite-Size Scaling above the Upper Critical Dimension

**DOI:** 10.3390/e26060509

**Published:** 2024-06-12

**Authors:** A. Peter Young

**Affiliations:** Physics Department, University of California, Santa Cruz, CA 95064, USA; petery@ucsc.edu

**Keywords:** phase transitions, finite-size scaling, hyperscaling, upper critical dimension

## Abstract

We consider how finite-size scaling (FSS) is modified above the upper critical dimension, $d_{u}=4$, due to hyperscaling violations, which in turn arise from a dangerous irrelevant variable. In addition to the commonly studied case of periodic boundary conditions, we also consider new effects that arise with free boundary conditions. Some numerical results are presented in addition to theoretical arguments.

## 1. Introduction

In the study of continuous phase transitions, finite-size scaling (FSS) is extensively used to extrapolate numerical results on a range of finite-size systems to the thermodynamic limit in order to determine properties of the transition, such as critical exponents. While the basic ideas are well established, some additional complications arise above the “upper critical dimension”, $d_{u}=4$, related to violations of hyperscaling relations (those relations between critical exponents which also involve the space dimension *d*) for bulk properties. In this article, we will discuss these additional features which arise for dimension d>4. For a recent review, see Ref. [1], which is by the late Ralph Kenna and collaborators.

The plan of the paper is as follows. In Section 2, we describe the theory of FSS and briefly indicate its justification from the renormalization group in Section 3. The new aspects of FSS above d=4 are given in Section 4. Section 4.1 explains how the new physics in this region is related to a “dangerous irrelevant variable”. Numerical results for periodic boundary conditions in d=4 are presented in Section 4.2, while a discussion of the finite-size correlation length is given in Section 4.3. The rather surprising new features that appear with periodic boundary conditions are explained in Section 4.4, and finally, we give a summary in Section 5.

## 2. Finite-Size Scaling

We know that a sharp phase transition can only take place in an infinite system, while for a finite system, the change in behavior at a transition is “rounded out” in some way. At a second-order transition, the correlation length ξ varies as

(1) $$\xi ∼{\left(T-T_{c}\right)}^{-\nu }\;,$$

where $T_{c}$ is the transition temperature, and ν is a critical exponent that characterizes the divergence of the correlation length. Consequently, close enough to $T_{c}$ that ξ>L, where *L* is the (linear) system size, finite-size corrections must be large.

Since numerical simulations are carried out on systems with finite sizes, in order to obtain accurate results in the thermodynamic limit, it is necessary to extrapolate results from several finite sizes to infinite sizes. The technique for doing this is called finite-size scaling (FSS). We will now outline the basic ideas of FSS, which were originally due to Michael Fisher [2,3].

We consider systems that are finite in all *d* dimensions, as in Monte Carlo simulations. One can also study other geometries in which one or more dimensions are infinite, the most important case being *one* infinite dimension, which corresponds to transfer matrix calculations and also to quantum systems at zero temperature. However, we will not consider that situation here. For simplicity, we assume that the system has the same size *L* in each of *d* dimensions, so the total size of the system is given by

(2) $$N=L^{d}.$$

We will consider models for magnetic materials in which a “spin” lies on each side of a hypercubic regular lattice and interacts with its neighbors. Most commonly, a spin might be a scalar that takes values $S_{i}=\pm 1$, which is known as the Ising model, but sometimes classical *m*-component vectors are taken instead. The examples considered here will all be for Ising models.

As soon as we move away from the thermodynamic limit and discuss finite-size systems, we have to specify the boundary conditions. In simulations, one would like finite-size corrections to be as small as possible, so normally, one considers period boundary conditions, where a site with coordinate L+1 in one of the directions is identified with site 1 in that direction. This means that there is no surface. Here, we will mainly consider periodic boundary conditions, but Section 4.4 will include the effects of surfaces by choosing free boundaries, in which a site on the surface has no coupling going outside the surface. One can convert periodic boundary conditions to free boundary conditions simply by setting to zero those interactions which “wrap around” the system.

When $T-T_{c}$ is sufficiently small that ξ≳L, the results of simulations will depend on *L*. By contrast, well above $T_{c}$, where L≫ξ, one expects properties of a system with periodic boundary conditions to differ by only exponentially small corrections of order exp(−L/ξ) from those of an infinite system. With free boundaries, where there is a surface, corrections will be much larger in this limit, typically of 1/L relative to the bulk values.

Let us consider some quantity *X* say, which diverges at $T_{c}$ as

(3) $$X_{\infty }\approx A{\left(T-T_{c}\right)}^{-\lambda }$$

in the thermodynamic limit. A simple postulate for how *X* might vary on a system of size *L* is

(4) $$\frac{X_{L}\left(T\right)}{X_{\infty }\left(T\right)}=f\left(\frac{L}{\xi }\right),$$

for some function f(x). One expects that *f* is universal in the renormalization group sense, i.e., it does not depend on the lattice structure or irrelevant variables. One does, however, expect that it will depend on the boundary conditions.

We can deduce the form of *f* in two limits:

1. At a fixed *T* in the limit L→∞ we have $X_{L}\to X_{\infty }$ so
  (5) $$\lim_{x\to \infty }f\left(x\right)=1.$$
2. At *L* fixed and $T\to T_{c}$, $X_{L}\left(T\right)$ is not singular because there can be no sharp transition in a finite system. This means that the behavior of f(x) for x→0 should compensate for the divergence of $X_{\infty }$, i.e.,
  (6) $$f\left(x\right)=Cx^{\lambda /\nu }\;\mathrm{for}\;x\to 0,$$
  where we used Equation (1).

In fact, $X_{L}\left(T\right)$ varies smoothly around $T=T_{c}$, and one can incorporate this, as well as have a single formula that works both above and below $T_{c}$, by rewriting Equation (4) as

(7) $$X_{L}\left(T\right)=L^{\lambda /\nu }\;\tilde{X}\left(L^{1/\nu }\;\left(T-T_{c}\right)\;\right),$$

where ignoring the amplitude *A* in Equation (3) for simplicity, one has

(8) $$\tilde{X}\left(x\right)=x^{-\lambda }f\left(x^{\nu }\right).$$

Since $X_{L}\left(0\right)$ is non-zero and finite, we must have $\tilde{X}\left(0\right)=\mathrm{const}.$, so Equation (7) gives a prediction for the size dependence at $T_{c}$, namely

(9) $$X_{L}\left(T_{c}\right)∼L^{\lambda /\nu }.$$

Equation (7) is the basic postulate of finite-size scaling. For what quantities *X* would we typically apply it? Naturally, we would like a quantity with a strong divergence, i.e., λ is greater than zero and not too small. An “obvious” choice is the susceptibility χ, which diverges with an exponent γ, which is exactly 7/4 in the two-dimensional Ising model and about 1.24 in the three-dimensional Ising model. However, determining χ is difficult in numerical simulations for the following reasons. It is computed from spin correlations using the relation

(10) $$\begin{array}{cc} \chi & =\frac{1}{T}\;\frac{1}{N}\sum_{i,j}\left[\langle S_{i}S_{j}\rangle -\langle S_{i}\rangle \langle S_{j}\rangle \right] \end{array}$$

(11) $$\begin{array}{cc} \; & =\frac{1}{T}N\left[\langle m^{2}\rangle -{\langle m\rangle }^{2}\right], \end{array}$$

where

(12) $$m=\frac{1}{N}\sum_{i=1}^{N}S_{i},$$

is the magnetization per site. However, for a finite system and in zero external magnetic fields, strictly speaking, one has 〈m〉=0. In practice, below $T_{c}$ the magnetization will very slowly fluctuate between the “up” state and the “down” state with a time that varies strongly with system size and temperature and may be much longer than the time of the simulation. Hence, at least below $T_{c}$, it is useful to calculate only quantities that are invariant under the global symmetry of the model. Here, for an Ising model in zero field, this is an inversion of all the spins.

Some proposed fixes for this problem are:

1. Replace the second term in Equation (11) by ${\langle |m|\rangle }^{2}$, i.e.,
  (13) $$\chi =\frac{1}{T}N\left[\langle m^{2}\rangle -{\langle |m|\rangle }^{2}\right],$$
  (the factor of 1/T is often omitted.) Equation (13) gives the desirable result that the two terms in Equation (11) tend to cancel below $T_{c}$, but unfortunately, it does not give the correct susceptibility above $T_{c}$. For example, in the limit T→∞, one has χ=(1−2/π)/T instead of the correct result, which is 1/T.
2. My preferred solution is to replace Equation (10) by
  (14) $$\chi =\frac{1}{T}\;\left[\frac{1}{N}\sum_{i,j}\langle S_{i}S_{j}\rangle -\sum_{i}\langle S_{i}S_{i+\vec{L}/2}\rangle \right],$$
  where $S_{i+\vec{L}/2}$ indicates the spin which is L/2 in every direction away from *i*, i.e., as far as possible from *i*. The second term is exponentially small in L/ξ above $T_{c}$, while below $T_{c}$ it tends to cancel the first term. Unfortunately, this definition of χ in a finite system has not been adopted.
3. Ignore the second term in Equations (10) and (11) completely. Typically, one also neglects the factor of 1/T since this varies only slightly in the vicinity of the transition. We will, therefore, define the correlation function
  (15) $$C=\frac{1}{N}\sum_{i,j}\langle S_{i}S_{j}\rangle =N\langle m^{2}\rangle .$$
  Well above $T_{c}$ this is the susceptibility, apart from the factor of 1/T, but below $T_{c}$ the dominant part varies as *N* times the magnetization squared, i.e., $N|T_{c}{-T|}^{2\beta }$ where β is the order parameter exponent defined by $\langle m\rangle ∼|T_{c}{-T|}^{\beta }$ for $T<T_{c}$. For χ this part is canceled by the second term in Equation (11). From Equation (7) we write
  (16) $$C=L^{2-\eta }\tilde{C}\left(L^{1/\nu }\left(T-T_{c}\right)\right),$$
  where we used the scaling relation
  (17) γ=(2−η)ν,
  in which the exponent η characterizes the power-law decay of correlations with distance at the critical point. The scaling function $\tilde{C}\left(x\right)$ must have the following limiting behaviors:
  (18) $$\tilde{C}\left(x\right)∼\left\{\begin{array}{cc} x^{-\gamma }, & \left(x\to \infty \right)\\ \mathrm{const}., & \left(x=0\right)\\ {\left|x\right|}^{2\beta }, & \left(x\to -\infty \right) \end{array}\right.,$$
  where, in the last line, we used the following “hyperscaling” scaling relation
  (19) $$\beta =\frac{1}{2}\left(d-2+\eta \right)\;\nu .$$
  Hyperscaling relations, which are the main focus of this volume, give connections between exponents which involve the space dimension *d*.

Equations (15)–(19) describe the FSS of the second moment of the order parameter *m*. Following Binder [4], it is of interest to consider other moments and, in particular, the whole distribution P(m). In order for the second moment to satisfy Equation (16) and for the distribution to be normalized, we must have

(20) $$P\left(m\right)=L^{\beta /\nu }\tilde{P}\left(L^{\beta /\nu }m,L^{1/\nu }\left(T-T_{c}\right)\right).$$

The power of *L* in front of the scaling function $\tilde{P}$ has to be the same as the power of *L* multiplying *m* in the first argument of $\tilde{P}$ in order that the distribution is normalized, and this power has to be β/ν in order to obtain Equation (16) (to see this note Equation (19)).

Binder [4] also pointed out that it is useful to look at ratios of moments such that the total power of *m* in the numerator and denominator are equal because then the power of *L* in front of the scaling function disappears. A simple and commonly studied case is the ratio of the fourth moment to the second moment squared,

(21) $$g=\frac{1}{2}\left(3-\frac{\langle m^{4}\rangle }{{\langle m^{2}\rangle }^{2}}\right),$$

which is known as the Binder ratio or Binder cumulant. The factors 3 and 1/2 are not essential but are often included so that *g* varies from 0 well above $T_{c}$ (since fluctuations there are Gaussian which implies $\langle m^{4}\rangle =3{\langle m^{2}\rangle }^{2}$) to 1 well below $T_{c}$ (since $\langle m^{4}\rangle ={\langle m^{2}\rangle }^{2}$ in that region). The FSS behavior of *g* is

(22) $$g=\tilde{g}\left(L^{1/\nu }\left(T-T_{c}\right)\right).$$

Later, we will consider the wavevector dependence of correlations so we generalize the second moment of the order parameter in Equation (15) to non-zero wavevectors,

(23) $$C\left(k\right)=\frac{1}{N}\sum_{i,j}e^{ik\cdot (R_{i}-R_{j})}\;\langle S_{i}S_{j}\rangle .$$

The standard FSS scaling form for C(k) for small k is the same as for k=0, namely Equation (16).

One reason the study of critical phenomena is particularly interesting is that many quantities are “universal”, i.e., they only depend on certain broad features of the problem, such as space dimensionality and the symmetry of the order parameter, but do not depend on microscopic details, such as the type of lattice and range of interactions (as long as they are not infinite range). Exponents are examples of universal quantities, while the transition temperature depends on all the microscopic details, and so does non-universal.

In addition, scaling functions, including finite-size scaling functions, have a degree of universality. Fisher and Privman [5] clarified the situation for FSS functions. Suppose we take Equation (7) for a general quantity $X_{L}$. There will be a non-universal amplitude (or metric factor) associated with the overall size of $X_{L}$ and a non-universal amplitude in front of the reduced temperature in the scaling function so we can write

(24) $$X_{L}\left(T\right)=AL^{\lambda /\nu }\;\tilde{X}\left(BL^{1/\nu }\left(T-T_{c}\right)\right),$$

where *A* and *B* are non-universal. According to Privman and Fisher [5], the resulting scaling function $\tilde{X}$ is universal, which means that it does not depend on microscopic details, but since we are dealing with finite-size effects, it does depend on the boundary conditions. If we include a magnetic field *h*, the scaling function has a second argument $CL^{y_{H}}h$. The amplitude *C* is non-universal, but the scaling function itself remains universal. Here, $y_{H}$ is called the magnetic exponent and is related to exponents introduced earlier by

(25) $$y_{H}=\frac{1}{2}\left(d+2-\eta \right)\;.$$

The exponent 1/ν, which is the power of *L* multiplying $T-T_{c}$ in the scaling functions, is called the thermal exponent and labeled $y_{T}$, so

(26) $$y_{T}=\frac{1}{\nu }.$$

**Figure 1:**
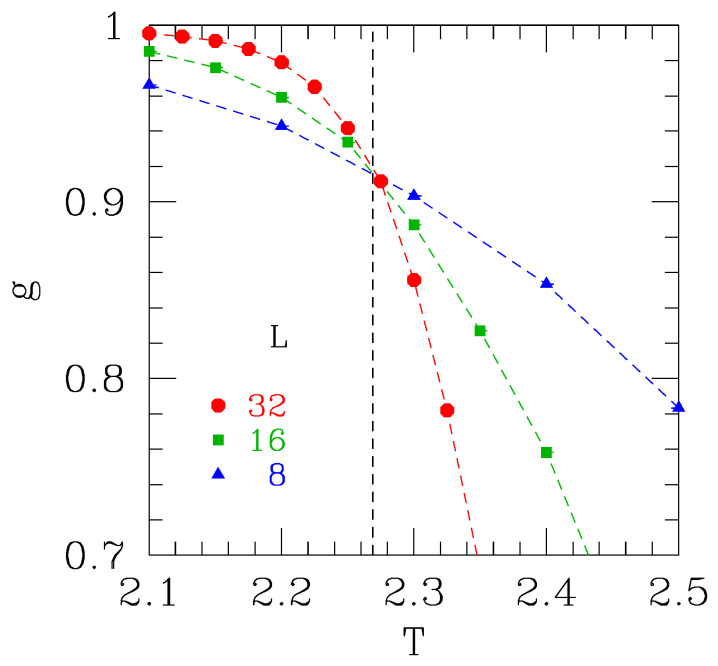
Calculation of the Binder ratio defined in Equation (21) for the two-dimensional Ising model on a square lattice with nearest-neighbor interactions. The dashed vertical line indicates the exact transition temperature $T_{c}=2/\ln\left(\sqrt{2}+1\right)=2.269\ldots$. The figure shows that $T_{c}$ is the temperature where the data for *g* for different sizes intersect. Furthermore, the *value* of *g* at the critical (intersection) point is of interest because it is universal. From more detailed calculations, this value is found to be 0.916 in three decimal places. Universality means that the same value would occur for a triangular lattice or a square lattice with second neighbor interactions, for example, even though the transition temperatures would be different for these models. The value of $g(T_{c})$ does, however, depend on the boundary conditions and is also different in different space dimensions.

An important application of FSS functions being universal occurs for dimensionless quantities such as the Binder ratio *g*. Equation (22) does not have a non-universal overall scale factor like *A* in Equation (24) multiplying the scaling function (since *g* is dimensionless), and so we write

(27) $$g=\tilde{g}\left(BL^{1/\nu }\left(T-T_{c}\right)\right)$$

with *B* non-universal and $\tilde{g}$ universal. Hence, the *value* of *g* at the intersection point (which is $T=T_{c}$) is a universal number; see Figure 1. Consequently, if the intersection values for two different systems known to be in the same universality class are apparently different, it follows that the asymptotic scaling regime has *not* been reached, and corrections to FSS are significant. This is a useful check.

## 3. Renormalization Group Justification for Finite-Size Scaling

To better understand FSS, we adopt a renormalization group (RG) perspective. We consider a system on a lattice with lattice spacing *a* with a Hamiltonian H which has interactions $K_{1},K_{2},\cdots$, into which we have absorbed the factor of $1/(k_{B}T)$. In the RG, we perform a coarse-graining of the system such that the new lattice spacing is ba with b>1, and the probability distribution of long wavelength fluctuations is unchanged. This distribution is governed by a new Hamiltonian $H^{\prime }$, of the same form as H but with new interactions $K_{1}^{\prime },K_{2}^{\prime },\cdots$.

If we are close to a second-order transition, the set of interactions will approach close to a fixed point $K_{1}^{*},K_{2}^{*},\cdots$. Near the fixed point is useful to consider linear combinations $u_{i}$ of the deviations $K_{\alpha }-K_{\alpha }^{*}$, which transform simply, $u_{i}^{\prime }=b^{y_{i}}u_{i}$, where $y_{i}$ is an eigenvalue of the RG transformation linearized about the fixed point. If $y_{i}>0$, then $u_{i}$ is said to be a “relevant” variable since iterating the RG transformation takes $u_{i}$ away from the fixed point ($u_{i}=0$), whereas if $y_{i}<0$, then $u_{i}$ is an “irrelevant” variable since $u_{i}$ decreases to zero upon iterating.

For a standard second-order transition, one has to set the temperature to the critical temperature and the magnetic field to zero in order to be at the critical point. Therefore, there are two relevant operators: the reduced temperature *t* defined by

(28) $$t=\frac{T-T_{c}}{T_{c}}$$

and the magnetic field *h*. One can show that the corresponding exponents, $y_{T}$ and $y_{H}$, are related to other exponents by Equations (25) and (26). In the following discussion, we will include just the most important irrelevant operator, i.e., the one with the least negative eigenvalue, and call it *u*. Following convention, we will write $y_{u}=-\omega$ where ω>0.

Under the RG transformation, quantities of interest are assumed to vary in the following way

(29) $$X\left(t,h,u,L\right)=b^{y_{X}}\;X\left(b^{y_{T}}y,\;b^{y_{H}}h,\;b^{-\omega }u,\;L/b\right),$$

where $y_{X}$ is related to the exponent, giving the divergence of *X* in the thermodynamic limit, as we shall see. If we fix h=0, ignore the irrelevant variable, and set b=L we obtain

(30) $$X_{L}\left(t\right)=L^{y_{X}}X\left(L^{y_{T}}t,0,0,1\right)=L^{y_{X}}\tilde{X}\left(L^{y_{T}}\;t\right),$$

which agrees with the basic FSS expression in Equation (7) with $y_{X}=\lambda /\nu =\lambda \;y_{T}$, where λ is the exponent for the divergence of *X* at the bulk critical point, see Equation (3).

Please note that we have assumed we can set the irrelevant variable *u* to zero to obtain Equation (7) and that including *u* would give corrections to scaling to that equation. However, we shall see that above the so-called upper critical dimension, $d_{u}=4$, some scaling functions develop singularities in this limit. In these cases, we cannot just set u=0 but need to consider the form of the singularity as *u* *tends* to 0. Such variables *u* are called “dangerous” irrelevant variables, a term first coined by Michael Fisher.

## 4. Finite-Size Scaling above the Upper Critical Dimension

### 4.1. Dangerous Irrelevant Variables

The simplest approximate theory to describe critical phenomena is mean-field theory (MFT), according to which the critical exponents we have mentioned so far have values

(31) γ=1,β=1/2,ν=1/2,η=0(MFT).

It is well established by the RG that MFT gives the exponents correctly for dimension *d* greater than the upper critical dimension d=4. However, for d<4, the exponents vary with *d*; for d>4, they “stick” at their MF values.

Earlier, we have mentioned two scaling relations, Equations (17) and (19), relating different exponents. Equation (17) relating γ and η *is* satisfied by their MF values. However, the hyperscaling relation in Equation (19) does *not* work for d>4. It does work, though, at the borderline dimension between MF and non-MF behavior, d=4, and so, for d>4, an equation analogous to Equation (19) with *d* replaced by $d_{u}\;\left(=4\right)$ *is* correct [6,7]. In this section, we will discuss the physics of the violation of hyperscaling relations in the mean-field regime for d>4 and show how this physics also gives rise to a form of FSS that is different from what we have discussed so far.

In RG treatments of critical phenomena, one starts with a “soft-spin” version of the problem, in which an initial coarse-graining over the discrete “fixed length” spins has been carried out. This Hamiltonian, associated with the names of Ginzburg, Landau, and Wilson, is, for scalar (Ising) spins,

(32) $$H_{GLW}=\int \;\left[\frac{1}{2}r\left(T\right)\phi^{2}+\frac{1}{2}{\left(\nabla \phi \right)}^{2}+\frac{1}{4}u\phi^{4}\right]d^{d}x,$$

where ϕ(x) is the “spin” variable, and the partition function is given by

(33) $$Z=\int D\left[\phi \left(x\right)\right]\exp\left(-H_{GLW}\right)\;.$$

The parameter r(T) varies smoothly with temperature and goes negative at low *T* in order to induce an ordering in ϕ. In this formulation, MFT corresponds to taking ϕ(x) to be independent of *x* and minimizing the resulting function with respect to ϕ. The transition temperature is when r=0, and at lower temperatures, one finds

(34) $$\langle \phi \rangle ={\left(\frac{\left|r\right|}{u}\right)}^{1/2}\;\left(\mathrm{MFT}\right)\;.$$

Since *r* varies smoothly with *T*, i.e., $r\left(T\right)\propto T-T_{c}$, we have $\langle \phi \rangle \propto {\left(T_{c}-T\right)}^{1/2}$ so β=1/2, the MF value that we quoted in Equation (31).

Renormalization group arguments show that for d>4, the quartic coupling *u* is irrelevant, so its value iterates to zero under repeated RG transformations. However, the expression for 〈ϕ〉 in Equation (34) becomes singular in this limit, so we cannot just set u=0. This is important because to derive the *standard* FSS result in Equation (7), we set the irrelevant variable to zero in Equation (30). However, for d>4, we cannot do this, just as we cannot set u=0 in Equation (34), but must rather consider the singular nature of the scaling function X(t,h,u,L) as *u tends* to 0. As mentioned in the previous section, irrelevant variables that give singularities if they are set to zero are said to be “dangerous”.

Hence Equations (7), (16) and (22), for example, are not valid for d>4. The way these equations have to be modified in the case of periodic boundary conditions was shown by Brézin and Zinn-Justin [8] and Binder et al. [9]. The argument of the standard FSS functions is $L^{1/\nu }\;\left(T-T_{c}\right)$. If we put the MF value ν=1/2, this would be $L^{2}\left(T-T_{c}\right)$. However Refs. [8,9] show that the correct result involves the square root of the volume, i.e., $L^{2}\left(T-T_{c}\right)$ is replaced by $L^{d/2}\;\left(T-T_{c}\right)$. The prefactor in front of the scaling function may also change in order to give the correct results for an infinite system. For d>4, the expressions corresponding to Equations (7), (16) and (22) are modified to

(35) $$X_{L}=L^{\lambda d/2}\tilde{X}\left(L^{d/2}\;\left(T-T_{c}\right)\right),\;C=L^{d/2}\tilde{C}\left(L^{d/2}\;\left(T-T_{c}\right)\right),\;g=\tilde{g}\left(L^{d/2}\;\left(T-T_{c}\right)\right),$$

Naively, one would expect that finite-size effects are substantial when the correlation length is of order *L*, which corresponds to $T-T_{c}∼L^{-2}$. However, Equation (35) shows that, for periodic boundary conditions, nothing happens at this temperature and finite-size effects only occur closer (Please note that we are considering d>4 so $L^{-d/2}<L^{-2}$). to $T_{c}$ when $T-T_{c}∼L^{-d/2}$. This surprising result is presumably due to (i) we have the periodic boundary conditions, which means there is no surface at a distance *L*, and (ii) the fixed point value of the coupling *u* in Equation (32) is zero, so long wavelength fluctuations interact very weakly.

### 4.2. Numerical Results for Periodic Boundary Conditions

It is possible to check the results in Equation (35) by Monte Carlo simulations on the five-dimensional Ising model. One can simulate large sizes using a clustering algorithm that was originally proposed by Swendsen and Wang [10] and subsequently modified by Wolff [11]. This algorithm considerably reduces the growth of relaxation times at the critical point (critical slowing down). Here, we will present some results found by Wittmann and the author [12].

The Hamiltonian is

(36) $$H=-\sum_{\langle i,j\rangle }J_{ij}S_{i}S_{j},$$

where the $S_{i}=\pm 1$ are Ising spins on a five-dimensional lattice of size $N=L^{5}$. In this subsection, we consider periodic boundary conditions, but in Section 4.4, we will consider free boundary conditions. The $J_{ij}$ are interactions between spins *i* and *j*, and we take $J_{ij}=1$ for nearest neighbors and 0 otherwise. Using the Wolff algorithm, it was possible to simulate sizes up to L=36.

**Figure 2:**
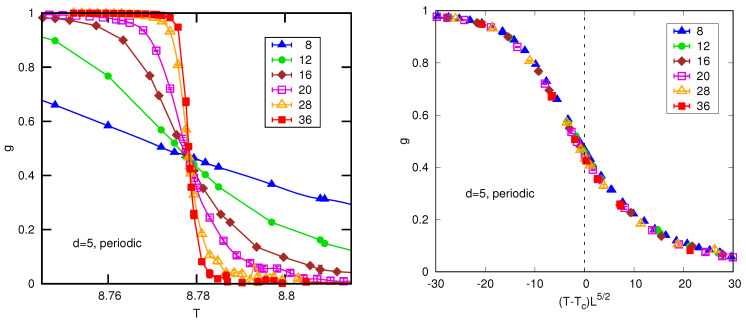
The left panel shows data for the Binder ratio *g* for the five-dimensional Ising model with periodic boundary conditions. The right panel shows the same data but scaled according to Equation (35), in which $T_{c}=8.77846$ and the power of *L* is d/2(=5/2 here), rather than 1/ν, the value expected in standard FSS (see Equation (22) which equals 2. The data collapse using the exponent 5/2 is seen to be good, whereas it is poor if the exponent value 2 is used. From Ref. [12].

Figure 2 shows data for the Binder ratio *g*, including a scaling plot according to Equation (35) with d=5, which works well. An attempt to scale the data in which the power of *L*, namely d/2, is replaced by 1/ν=2, as in standard FSS (see Equation (22)), is very poor.

We emphasize that the reason we have the power d/2 rather than 2 in Equation (35) ultimately comes from Equation (34), namely that the order parameter 〈ϕ〉 is singular for u→0. It is, therefore, interesting to ask what form FSS would take if we look at some quantity that does not involve the order parameter (which is a k=0 quantity).

**Figure 3:**
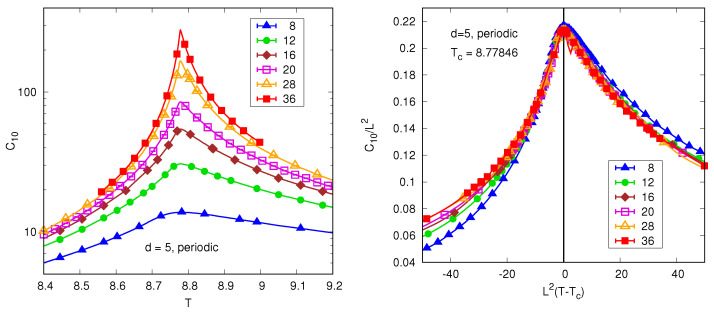
The left panel shows data for the wavevector dependent correlation function C(k) for k=(2π/L)(1,0,0,0,0) for the five-dimensional Ising model with periodic boundary conditions. The right panel shows the data scaled according to the standard FSS form in Equation (16) using mean-field exponents ν=1/2,η=0, rather than the modified FSS form in Equation (35) with d=5. This scaling works well apart from the smallest size, L=8, especially above $T_{c}$.

Ref. [12] investigated this question numerically by computing the wavevector dependent correlation function C(k) in Equation (23) for some small but non-zero wavevectors, which, we note, are *orthogonal* to the order parameter since we still consider periodic boundary conditions. Figure 3 shows results for C(k) where k=(2π/L)(1,0,0,0,0), the smallest non-zero wavevector. The data increases as *T* decreases to $T_{c}$ but then decreases below $T_{c}$ because these fluctuations are orthogonal to the order parameter. Apart from the smallest size presented (L=8), the data scales well but with the standard FSS exponent form, Equation (16) with ν=1/2,η=0, rather than the modified FSS form in Equation (35) with d=5.

A natural hypothesis, then, is that *all* quantities which do not involve the order parameter satisfy standard FSS expressions even for d>4.

### 4.3. The Finite-Size Correlation Length

The divergence of the bulk correlation length at $T_{c}$ is an important feature of a second-order transition, so it is of interest to define the correlation length in a finite system as well.

The correlation length of the finite system is given by the following finite difference expression [13,14],

(37) $$\xi_{L}=\frac{1}{2\sin(k_{\min}/2)}\sqrt{\frac{C(0)}{C(k_{min})}-1}\;,$$

where $k_{min}$ is the smallest non-zero wavevector on the lattice. For example, in d=5, $k_{min}=\left(2\pi /L\right)\left(1,0,0,0,0\right)$ and permutations. In this section, we continue to assume periodic boundary conditions. With this definition of $\xi_{L}$, the correlation function far enough above $T_{c}$ obeys the expected Ornstein–Zernicke form

(38) $$C\left(k\right)=\frac{C(0)}{1+\xi_{L}^{2}k^{2}+\cdots },$$

so $\xi_{L}$ is indeed a characteristic length in this region. However, it can not be interpreted as a physical length below $T_{c}$ since C(0) is dominated by the long-range order and so diverges as $L^{d}$, whereas $C(k_{\min})$ does not have a long-range order component and so is finite below $T_{c}$. Nonetheless, $\xi_{L}$ is a convenient quantity to analyze with FSS in *all* regions since, like the Binder ratio *g*, $\xi_{L}/L$ is dimensionless, and so its FSS form does not have the power of *L* multiplying the scaling function.

**Figure 4:**
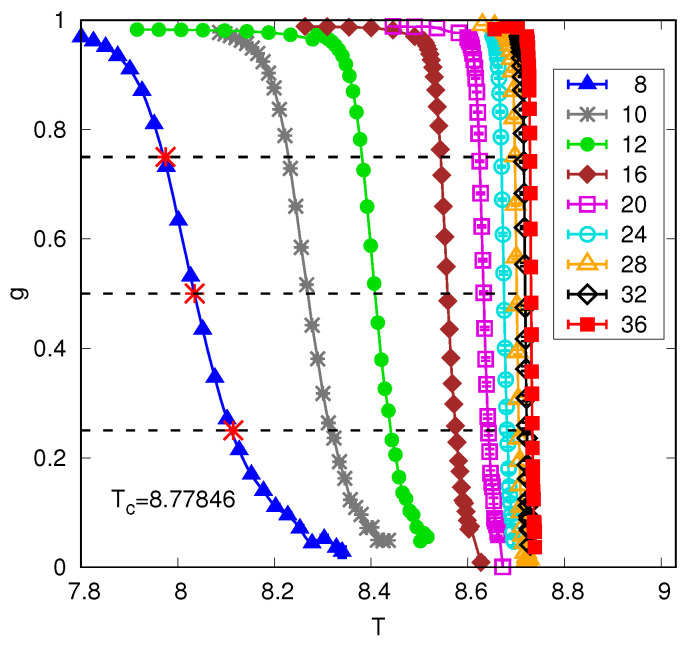
The Binder ratio defined in Equation (21) for the five-dimensional Ising model with free boundary conditions. The data are from Ref. [12]. The region of finite-size rounding, $\delta t_{L}$, is defined here, for each size, to be the range of temperature between where *g* has values 0.75 and 0.25. For example, for L=8 we have $\delta t_{8}=8.115-7.975=0.140$ (these points are indicated by crosses). The shift $\Delta T_{L}$ is defined here to be the difference between $T_{c}\left(=8.77846\right)$ and the value of *T* where g=0.5. For L=8, we find g=0.5 at T=8.035 (shown as a cross in the figure), so, taking $T_{c}=8.77846$ from data on periodic boundary conditions, $\Delta T_{8}=8.77846-8.035=0.743$. Analysis of the data shows that the rounding tends to zero as $1/L^{5/2}$ while the shift tends to zero as $1/L^{2}$, which is bigger.

According to [12], for k>0 but kL≪1, C(k) varies as $L^{2}$ at $T_{c}$, see Figure 4. This indicates that in real space, correlations fall off with distance in the expected mean-field way, namely $1/r^{d-2}$ so η=0. However C(0) goes as $L^{d/2}$ which is much bigger than $C(k_{min})$ for d>4. Hence, as defined in Equation (37), we see that for large *L*

(39) $$\xi_{L}\propto L{\left(\frac{L^{d/2}}{L^{2}}\right)}^{1/2}=L^{d/4}$$

at $T_{c}$ which is bigger than *L* since d>4 here. However, in contrast to some earlier results [7,14], we argue that we should not interpret $\xi_{L}$ as a physical length *at* $T_{c}$. Rather the quantity defined to be $\xi_{L}$ is larger than *L* at $T_{c}$ only because C(0) is anomalously large relative to C(k>0). To be more precise, correlations fall off with distance in the expected mean-field way, i.e., as $1/r^{d-2}$, so one would naively think that at a distance of order L/2, the correlations would be of order $1/L^{d-2}$. However, this is wrong because the k=0 mode gives an additional, anomalously large, constant contribution of order $1/L^{d/2}$.

Finally, we note that the FSS form for $\xi_{L}$ defined in Equation (37) is [14]

(40) $$\xi_{L}=L^{d/4}\tilde{\xi }\left(L^{d/2}\left(T-T_{c}\right)\;\right),$$

which gives the $L^{d/4}$ behavior at $T_{c}$ derived earlier in Equation (39).

### 4.4. Free Boundary Conditions above the Upper Critical Dimension

The above results are for periodic boundary conditions for d>4, and they show that the temperature range over which there is finite-size rounding is of order $1/L^{d/2}$. This means that there is no change in behavior when ξ∼L, i.e., $T-T_{c}∼L^{-2}$, but only closer to $T_{c}$ when $T-T_{c}∼L^{-d/2}$. While surprising, this result is nonetheless possible with periodic boundary conditions. However, if we have free boundary conditions, surely *something* must happen when ξ∼L because the correlations must be affected by the surfaces.

To our knowledge, the question of what happens with free boundary conditions for d>4 was first addressed by Rudnick et al. [15]. They showed that one needs to consider two exponents describing finite-size effects: a “shift” exponent and a “rounding” exponent. The possibility of two exponents to characterize finite-size effects was further studied by [12], who performed numerics on the five-dimensional Ising model with free boundary conditions and also considered possible different behavior for quantities that are orthogonal to the order parameter compared with those that involve the order parameter.

More precisely, ref. [12] computed the Fourier-transformed correlation functions

(41) $$C\left(k\right)=L^{d}{\langle |m\left(k\right)|}^{2}\rangle \;,$$

in which the Fourier-transformed magnetization, m(k), is defined differently for free boundary conditions compared with periodic boundary conditions. However, for periodic boundary conditions, the Fourier modes are plane waves, i.e.,

(42) $$m\left(k\right)=\frac{1}{N}\sum_{i}e^{ik\cdot r_{i}}\;S_{i},\;\;\left(\mathrm{periodic}\right),$$

where

(43) $$k_{\alpha }=2\pi n_{\alpha }/L,\;\;\left(\mathrm{periodic}\right),$$

with $n_{\alpha }=0,1,\cdots ,L-1$ and α denotes a Cartesian coordinate, for free boundary conditions, the Fourier modes are sine waves,

(44) $$m\left(k\right)=\frac{1}{N}\sum_{i}\left[\;\prod_{\alpha =1}^{d}\sin\left(k_{\alpha }r_{i,\alpha }\right)\;\right]S_{i},\;\;\left(\mathrm{free}\right),$$

where

(45) $$k_{\alpha }=\pi n_{\alpha }/\left(L+1\right),\;\;\left(\mathrm{free}\right),$$

with $n_{\alpha }=1,2,\cdots ,L$ and the components of the lattice position, $r_{i,\alpha }$, run over values 1,2,⋯,L. There is zero contribution to the sum in Equation (44) if we set $r_{i,\alpha }=0$ or L+1, so Equations (44) and (45) correctly incorporate free boundary conditions.

Please note that k=0 is not an allowed wavevector with free boundary conditions, so the uniform magnetization does not correspond to a single Fourier mode. Wavevectors with all $n_{\alpha }$ odd have a projection onto the uniform magnetization and so will acquire a non-zero expectation value below $T_{c}$ in the thermodynamic limit. It is, therefore, natural to expect that they will obey some sort of non-standard FSS. However, if any of the $n_{\alpha }$ is even, there is no projection onto the uniform magnetization, so they will not acquire an expectation value below $T_{c}$, and hence it is expected that they will satisfy standard FSS, i.e., Equation (16) with η=0, ν=1/2.

Ref. [12] found that corrections to FSS are larger with free boundary conditions than with periodic boundary conditions. Nonetheless, given the large sizes that can be studied with the Wolff algorithm, ref. [12] concluded that the data shows fairly convincingly that quantities that are orthogonal to the order parameter do indeed follow *standard* FSS.

What, then, is the form of non-standard FSS above the upper critical dimension with free boundary conditions for quantities that involve the order parameter? As already noted above, Rudnick et al. [15] showed that one needs both a temperature “shift” and a different temperature “rounding”. For the shift, one defines for each size a characteristic temperature (T(L) is called a pseudocritical temperature by Berche et al. [6], and we shall use that terminology here. In that paper, T(L) is determined from the maximum of χ computed with the subtracted term shown in Equation (13), rather than from the midpoint value of *g* as here. Any reasonable definition of T(L) should give the same exponent for the shift). T(L) from which a shift exponent λ is obtained as follows:

(46) $$\Delta T_{L}\equiv T_{c}-T\left(L\right)=\frac{A}{L^{\lambda }}.$$

The definition of T(L) is not unique, but one convenient choice, the one used by [12], is the temperature where g(T(L))=0.5, see Figure 4. A convenient choice for the temperature rounding is the difference between the temperatures where g=0.25 (say) and g=0.75 (say), i.e.,

(47) $$\delta t_{L}\equiv T\left(g=0.25\right)-T\left(g=0.75\right)=\frac{B}{L^{\mu }},$$

see Figure 4. This defines the rounding exponent μ.

In the presence of distinct exponents for shift and rounding, FSS expressions are the same as before, except that $T-T_{c}$ is replaced by T−T(L) so, for *g* for example, we have

(48) $$g=\tilde{g}\left(\;L^{\mu }\;\left(T-T\left(L\right)\right)\;\right)=\tilde{g}\left(\;L^{\mu }\;\left(T-T_{c}\right)+AL^{\mu -\lambda }\;\right),$$

rather than Equation (21). Please note that it is only necessary to consider the shift separately from the rounding if the size of the temperature shift is greater than the size of the temperature rounding, i.e., if λ<μ. Suppose λ>μ, the effects of the shift can be regarded as a correction to scaling since $AL^{\mu -\lambda }\to 0$ for L→∞ in this case. If λ=μ, one can view Equation (48) as usual FSS but with an unimportant shift in the origin of the scaling function $\tilde{g}\left(x\right)$.

Rudnick et al. [15] showed that

(49) $$\lambda =\frac{1}{\nu }=2,\;\mu =\frac{d}{2}\;\left(=\frac{5}{2}\;\mathrm{here}\right),$$

so Equation (48) can be written as

(50) $$g=\tilde{g}\left(\;L^{5/2}\;\left(T-T_{c}\right)+AL^{1/2}\;\right).$$

Figure 4 shows the numerical results of [12] in five dimensions with free boundary conditions. One can see by eye that the shift is bigger than the rounding, and analysis confirms the values given in Equation (49), namely a shift exponent of 2 and a rounding exponent of 5/2. These exponent values were also found numerically by Berche et al. [6].

Please note that at the critical point, the argument of the scaling function for *g* tends to infinity, where g=0. Hence, the data for different sizes do not intersect at $T_{c}$ for free boundary conditions and d>4, unlike the case for periodic boundary conditions, see Figure 2, and quite generally for d<4, see Figure 1.

It is also interesting to discuss the correlation function *C* defined in Equation (15), which serves as a proxy for the susceptibility χ. Taking the FSS form in Equation (35), which would be valid above the upper critical dimension for periodic boundary conditions, and replacing $T_{c}$ by the pseudocritical temperature T(L) to make it valid for free boundary conditions, we get

(51a) $$\begin{array}{cc} C & =\;L^{d/2}\tilde{C}\left(L^{d/2}(T-T\left(L\right)\;\right), \end{array}$$

(51b) $$\begin{array}{cc} \; & =\;L^{d/2}\tilde{C}\left(L^{d/2}\left(T-T_{c}\right)+AL^{d/2-2}\right). \end{array}$$

According to Equation (51a), at the pseudocritical temperature T(L), *C* diverges as $L^{d/2}$. Berche et al. [6] computed the susceptibility with a subtracted term as in Equation (13), which is expected to have the same scaling behavior as *C*, and found the predicted $L^{d/2}$ behavior.

Interestingly, the behavior at the true $T_{c}$ is different. Since the susceptibility diverges with an exponent γ=1 in the mean-field region, the scaling function $\tilde{C}\left(x\right)$ must vary as 1/x for x→∞. Since $T=T_{c}$ corresponds to this large *x* limit, it follows from Equation (51b) that the behavior of *C* at $T_{c}$ has the form

(52) $$C\left(T_{c}\right)\propto L^{d/2}\frac{1}{L^{d/2-2}}=L^{2}.$$

A strict upper bound shows that $C(T_{c})$ cannot be greater than $L^{2}$; see Refs. [16,17]. It seems, then, that this upper bound is satisfied as an equality. However, numerically, the situation is not very clear because there are large corrections to scaling due to a substantial fraction of sites being on the surface. Berche et al. [6] found an exponent of 1.71±0.02 if all sites are included in the analysis. However, this changed substantially to 1.92±0.02 if the outer half of the sites is removed when doing the average. Reference [6] claims that this value is incompatible with the above value of 2. However, this conclusion is not obvious to us since the error bar presumably includes only statistical errors, and very likely, some systematic errors remain for the sizes studied. Hence, in our view, the numerics of Ref. [6] does not rule out that *C* (or χ) varies like $L^{2}$ at $T_{c}$ with free boundary conditions. We also note that Lundow et al. [18] find that $\chi \propto L^{2}$ at $T_{c}$ for the 5d Ising model at $T_{c}$.

## 5. Conclusions

We summarize our understanding of FSS above and below the upper critical dimension, $d_{u}=4$ as follows:

- *Below the upper critical dimension.*
  Here, standard FSS applies:
  (53) $$\begin{array}{ccc} X_{L} & =\;L^{\lambda /\nu }\tilde{X}\left(L^{1/\nu }\;\left(T-T_{c}\right)\;\right),\;\;\; & C=L^{2-\eta }\tilde{C}\left(L^{1/\nu }\;\left(T-T_{c}\right)\;\right),\\ g & =\;\tilde{g}\left(L^{1/\nu }\;\left(T-T_{c}\right)\;\right),\; & \xi_{L}=L\;\tilde{\xi }\left(L^{1/\nu }\;\left(T-T_{c}\right)\;\right). \end{array}$$
  We recall that *C* is the k=0 correlation function in Equation (15) (a proxy for the susceptibility χ), *g* is the Binder ratio defined in Equation (21), $\xi_{L}$ is the quantity defined in Equation (37) which above $T_{c}$ is the correlation length, and *X* is a generic quantity which diverges like ${\left(T-T_{c}\right)}^{-\lambda }$ for $T\to T_{c}$.
- *Above the upper critical dimension with periodic boundary conditions.*
  For quantities that involve uniform magnetization, FSS scaling is modified to
  (54) $$\begin{array}{ccc} X_{L} & =\;L^{\lambda d/2}\tilde{X}\left(L^{d/2}\;\left(T-T_{c}\right)\;\right),\;\; & C=L^{d/2}\tilde{C}\left(L^{d/2}\;\left(T-T_{c}\right)\;\right),\\ g & =\;\tilde{g}\left(L^{d/2}\;\left(T-T_{c}\right)\;\right),\;\; & \xi_{L}=L^{d/4}\;\tilde{\xi }\left(L^{d/2}\;\left(T-T_{c}\right)\;\right). \end{array}$$
  For quantities that are orthogonal to the uniform magnetization, such as C(k) for k≠0) (but kL≪1), we have standard FSS with mean-field exponents, so
  (55) $$C\left(k\right)=L^{2}\tilde{C}\left(L^{2}\;\left(T-T_{c}\right)\;\right).$$
  These results imply that the correlation function at the critical point varies according to Gaussian behavior, i.e., $\langle S_{i}S_{j}\rangle ∼1/r_{ij}^{d-2}$ for $r_{ij}\equiv \left|r_{i}-r_{j}\right|\ll L/2$ but this is superimposed on a uniform background contribution (which only affects the k=0 mode) of order $1/L^{d/2}$. This was stated explicitly in Ref. [19], who demonstrated it numerically for the self-avoiding walk-in d=5, which is expected to be in the same universality class as the Ising model above the upper critical dimension.
- *Above the upper critical dimension with free boundary conditions.*
  For most quantities, we need to define a shift and a rounding. The shift exponent is 1/ν(=2), and the rounding exponent is d/2 (the same as for periodic boundary conditions). Please note that the temperature range due to the shift is bigger than the temperature range due to rounding. Defining a pseudocritical temperature T(L) for each size, most quantities behave as in Equation (54) but with $T_{c}$ replaced by T(L), i.e.,
  (56) $$\begin{array}{ccc} X_{L} & =\;L^{\lambda d/2}\tilde{X}\left(L^{d/2}\;\left(T-T\left(L\right)\right)\;\right),\;\; & C=L^{d/2}\tilde{C}\left(L^{d/2}\;\left(T-T\left(L\right)\right)\;\right),\\ g & =\;\tilde{g}\left(L^{d/2}\;\left(T-T\left(L\right)\right)\;\right),\;\; & \xi_{L}=L^{d/4}\;\tilde{\xi }\left(L^{d/2}\;\left(T-T\left(L\right)\right)\;\right), \end{array}$$
  where T(L) is related to $T_{c}$ by Equation (46) with λ=2. However, as discussed in Section 4.4, some of the normal modes are orthogonal to the uniform magnetization, and for these, only the exponent 1/ν(=2) appears and their correlations behave as in Equation (55).

The above conclusions are supported by theoretical arguments and numerical simulations. One of our main points is that for d>4 and periodic boundary conditions, the new features of FSS, which come from hyperscaling violation and the dangerous irrelevant variable, only manifest themselves for k=0 fluctuations. We note that in the simulations, corrections to the asymptotic behavior are quite large, especially for free boundary conditions, so the numerical estimates of exponents have errors. Indeed, other authors, in particular Refs. [6,7], have a somewhat different point of view from that presented here. For example, Ref. [7] argues that one should define two η-like exponents for periodic boundary conditions. However, we do not feel that this is necessary to understand our numerical results.

Finally, I will mention some more recent work. A central result in the theory of FSS for $d>d_{u}$ is that a complete description needs both the standard FSS exponent 1/ν, which is 2 here and the hyperscaling violation exponent d/2. An interesting and different way of understanding why both exponents are needed is discussed by Fang et al. [20]. In later work, Fang and collaborators [21] show that a complete description of FSS above the upper critical dimension is obtained by considering the Fortuin–Kasteleyn cluster representation of the states of the Ising model. These clusters show a change in behavior not only at $d_{u}=4$ but also at d=6.

In a recent development, Ref. [22] has shown that similar ideas to those reviewed here also apply to the random field Ising model, which has a different upper critical dimension, $d_{u}=6$, as opposed to $d_{u}=4$ for the more usual situation discussed here.

As a final remark, I should mention that I was invited to contribute to this Special Issue by Ralph Kenna, who sadly has since passed away. I never met Ralph, but was familiar with his work on finite-size scaling, which always contained original and stimulating ideas. I am pleased that his long-time collaborator, Bertrand Berche, has agreed to take over Ralph’s role of editor and bring this Special Issue to completion.

## Data Availability

The data presented in this study are available on request from the corresponding author.
